# A parainfluenza virus 5 (PIV5)-vectored intranasal SARS-CoV-2 vaccine (CVXGA1) elicits protective and long-lasting immunity in nonhuman primates

**DOI:** 10.1128/jvi.01990-24

**Published:** 2025-03-21

**Authors:** Ashley C. Beavis, Peng Xiao, Maria Cristina Gingerich, Kelsey Briggs, Geng Li, Elizabeth W. Howerth, Maria Najera, Dong An, Jiachen Huang, Jarrod Mousa, S. Mark Tompkins, Gina Kim, Stephen B. Harvey, Robert Jeffrey Hogan, Eric R. Lafontaine, Francois J. Villinger, Hong Jin, Biao He

**Affiliations:** 1 CyanVac LLC, Athens, Georgia, USA; 2 Department of Infectious Diseases, College of Veterinary Medicine, University of Georgia 551782 , Athens, Georgia, USA; 3 New Iberia Research Center, University of Louisiana at Lafayette 4365 , Lafayette, Louisiana, USA; 4 Department of Pathology, College of Veterinary Medicine, University of Georgia 70734 , Athens, Georgia, USA; 5 Animal Resources, University of Georgia 1355 , Athens, Georgia, USA; 6 Department of Population Health, College of Veterinary Medicine, University of Georgia 308501 , Athens, Georgia, USA; St. Jude Children's Research Hospital, Memphis, Tennessee, USA

**Keywords:** CVXGA1, SARS-CoV-2, viral-vectored vaccine, parainfluenza virus 5 (PIV5), African green monkey, nonhuman primate

## Abstract

Waning immunity from approved COVID-19 vaccines and emerging variants of SARS-CoV-2 necessitate the need to develop alternative COVID-19 vaccines for improved durability and broader protection. This work investigates the efficacy and mucosal, humoral, and cellular immunogenicity of intranasal, parainfluenza virus 5 (PIV5)-vectored COVID-19 vaccine CVXGA1 in an African green monkey (AGM) nonhuman primate model. A single intranasal dose of CVXGA1 induced robust and sustained humoral and cellular immune responses in AGMs and protected against SARS-CoV-2 ancestral WA1 strain and alpha variant challenge infection in the respiratory tracts as demonstrated by lack of viral load and greatly decreased histopathology. Mucosal IgA antibodies were detected in the upper and lower respiratory tracts of immunized AGMs. CVXGA1-vaccinated AGMs maintained serum anti-S IgG and IgA antibody titers for over 245 days. S-specific CD4^+^ and CD8^+^ T cells producing predominantly IFN-γ, TNF-α, MIP-β, and CD107α cytokines peaked on day 28 and could be detected on day 180 by intracellular cytokine staining of peripheral blood mononuclear cells (PBMC). IL13-secreting CD4^+^ and CD8^+^ T cells were minimally detected, indicative of a dominant type 1 immune response. These data supported the clinical evaluation of CVXGA1 intranasal COVID-19 vaccine. These data demonstrate that intranasal immunization with CVXGA1 induces long-lasting protective SARS-CoV-2 S-specific mucosal, humoral, and cellular responses in AGMs.

**IMPORTANCE:**

The continued threat of SARS-CoV-2 indicates the need for a novel vaccine that induces long-lasting mucosal, cellular, and humoral immunity, as well as block transmission. This work demonstrates that intranasal, PIV5 viral-vectored SARS-CoV-2 vaccine CVXGA1 induces mucosal immunity and long-lasting cellular and humoral immunity that protects African green monkeys from SARS-CoV-2 challenge. The ability of our intranasal vaccine to elicit long-lasting mucosal, cellular, and humoral immunity against SARS-CoV-2 indicates great promise of the PIV5-vectored COVID-19 vaccine for further clinical development.

## INTRODUCTION

SARS-CoV-2 is the causative agent of the COVID-19 pandemic (1) and has caused more than 760 million infections and 6.9 million deaths (2). In the United States, four SARS-CoV-2 vaccines are currently approved for use: mRNA vaccines developed by Pfizer and Moderna, a human adenovirus 26-vectored vaccine developed by Johnson & Johnson, and a subunit vaccine developed by Novavax (3, 4). More than 13 billion doses of SARS-CoV-2 vaccines have been administered throughout the world (2). However, waning and limited mucosal immunity leaves vaccinated individuals susceptible to infection (5). A novel vaccine capable of inducing mucosal, long-lasting, and protective immunity against SARS-CoV-2 transmission is still urgently needed. To address this, we generated an intranasal, parainfluenza virus 5 (PIV5)-vectored vaccine expressing the spike (S) protein from SARS-CoV-2 (CVXGA1) and tested its immunogenicity and efficacy in a nonhuman primate model.

The African Green Monkey (AGM) model is a well-established animal model to evaluate vaccine-mediated immune responses and vaccine efficacy against SARS-CoV-2 infection. Following SARS-CoV-2 infection, infectious virus can be isolated from AGM nasal wash (NW) and bronchoalveolar lavage (BAL) (6). Although clinical symptoms are infrequent, some older AGMs present human-like COVID-19 symptoms, including acute respiratory distress (ARD) and lung damage (7). Finally, AGMs can develop SARS-CoV-2 neutralizing antibodies and viral-specific cellular immune responses (6), making it an ideal model for testing SARS-CoV-2 vaccine candidates prior to clinical trials.

PIV5 is a negative-sense, single-stranded RNA virus in the family *Paramyxoviridae*, and its genome has 15,246 nucleotides that encode eight proteins (8, 9). PIV5 has been used as a vector to develop intranasal vaccines against numerous respiratory viruses including influenza, respiratory syncytial virus (RSV), MERS-CoV, and SARS-CoV-2 (10–13). An intranasal PIV5-vectored RSV vaccine-induced antigen-specific cellular and mucosal immunity in nonhuman primates (14), was proven safe in phase 1 clinical trial, and was approved for a phase 1/2 clinical trial in infants (15). A single, intranasal dose of CVXGA1 protected ferrets from SARS-CoV-2 challenge and transmission (13). CVXGA1 has been shown to elicit protective immune responses in several small animal models and block contact transmission in ferrets, but its ability to elicit SARS-CoV-2-specific cellular and mucosal immunity has not been well investigated. In this work, we investigated the longevity and protective efficacy of CVXGA1 and demonstrated the ability of CVXGA1 to induce mucosal antibodies and multi-functional cellular responses in AGMs.

## MATERIALS AND METHODS

### Cells and viruses

Vero E6 cells were maintained in Dulbecco’s modified Eagle medium (DMEM) supplemented with 5% fetal bovine serum (FBS), 100 IU/mL penicillin, and 100 µg/mL streptomycin (1% P/S; Mediatech Inc, Manassas, VA, USA). Serum-free (SF) Vero cells were maintained in VP-SFM (ThermoFisher Scientific) supplemented with 4 mM GlutaMax (Gibco). All cells were incubated at 37°C with 5% CO_2_.

CVXGA1 vaccine virus with SARS-CoV-2 S gene inserted into PIV5 genome has been described previously (13). SARS-CoV-2 WA1 strain was obtained from BEI Resources (catalog #NR-52281).

### African green monkey (AGM) immunization and challenge

AGMs were wild-caught from St. Kitts, housed in animal biosafety level (ABSL)−2 facilities at the New Iberia Research Center of the University of Louisiana at Lafayette, and maintained in accordance with the rules and regulations of the Committee on the Care and Use of Laboratory Animal Resources.

AGM study 1 evaluated CVXGA1 for antibody responses and its protection against SARS-CoV-2 challenge. AGMs were anesthetized with ketamine HCl and received a single, intranasal dose of 10^6^ PFU CVXGA1 (*n* = 4). Unvaccinated monkeys were used as negative controls (“Mock,” *n* = 4). Blood was collected on days −7, 14, 28, and 36 post-immunization. Nasal swabs were collected at days −7 and 28 post-immunization. At day 36 post-immunization, AGMs were challenged with SARS-CoV-2 WA1 strain in the ABSL-3 facility at UGA. Nasal wash samples were collected on days 1, 3, 5, 7, and 15 or 16 (15/16) post-challenge. BAL samples were collected at days 1, 5, and 15/16 post-challenge.

AGM study 2 evaluated CVXGA1 for protection and challenge virus tissue distribution. AGMs were anesthetized with ketamine HCl and received a single, intranasal dose of 10^6^ PFU CVXGA1 (*n* = 4). Unvaccinated monkeys were used as negative controls (“Mock,” *n* = 4). At day 28 post-immunization, AGMs were challenged with SARS-CoV-2 WA1. AGMs were euthanized under deep anesthesia at 2 or 4 days post-challenge with pentobarbital euthanasia solution IV, and the following tissues were collected: left lung, right lung, accessory lobe, nasal turbinate, trachea, and lymph node.

AGM study 3 was divided into two sub-studies, A and B. Study 3A evaluated CVXGA1 for protection against heterologous challenge with alpha variant. AGMs were anesthetized with ketamine HCl and received a single intranasal immunization of 10^6^ PFU vector control (BLB-201, “Vector control”) or CVXGA1 (*n* = 4). At 40 days post-immunization, AGMs were challenged with SARS-CoV-2 alpha variant. For each group, 2 AGMs were euthanized on days 3 and 6 post-challenge, and the following tissues were collected: nasal mucosa, trachea, tracheobronchial lymph node, heart, tongue, spleen, ileum, colon, and sections from all seven lung lobes. Tissues were collected into 10% formalin, paraffin-embedded, and 4 µm sections were stained with hematoxylin and eosin (HE) or for SARS-CoV-2 immunohistochemistry (IHC). Study 3B evaluated the longevity of antibody and cellular immune responses and boosting effect. AGMs were anesthetized with ketamine HCl and received 10^6^ PFU BLB-201, 10^5^ PFU CVXGA1, 10^6^ PFU CVXGA1, or 10^7^ PFU CVXGA1 (*n* = 4). AGMs who received 10^6^ PFU CVXGA1 were boosted with a second intranasal dose of 10^6^ PFU CVXGA1 at day 28 post-prime. Blood was collected at days −1, 7, 14, 28, 42, 56, 70, 180, and 245 post-immunization.

Before challenge with SARS-CoV-2 WA1, the AGMs were transferred to an ABSL3 facility at the University of Georgia (UGA) and housed in compliance with state and federal guidelines, the UGA Institutional Biosafety Committee (a program accredited by the Association for Assessment and Accreditation for Laboratory Animal Science, International), and the UGA Institutional Animal Care and Use Committee. For challenge infection with SARS-CoV-2 virus, AGMs were anesthetized via intramuscular injection of 4–8 mg/kg tiletamine-zolazepam. SARS-CoV-2 WA1 challenge virus (0.5 mL at 4 × 10^6^ PFU/mL) was delivered to each nostril (0.25 mL/nostril) using a Teleflex MAD nasal mucosal atomization device (Teleflex).

### AGM sample collection

AGMs were anesthetized or euthanized for sample collections. Blood was collected from femoral, cephalic, or saphenous veins. For mucosal fluid collections, a sterile sponge was moistened with 0.05 mL saline, inserted into the AGM nostril, and gently moved against the mucosa for 2 min to collect swabs from both nostrils and added to 2 mL microcentrifuge tubes. The samples were centrifuged at 400 × *g* for 10 min, and the supernatants were frozen at −80°C. To collect bronchoalveolar lavage (BAL) samples, anesthetized AGMs received approximately 10 mL 0.9% isotonic pharmaceutical-grade saline instilled into a bronchus via an AirLife Tri-Flo Suction Catheter passed through the endotracheal tube and immediately re-aspirated (16). The BAL samples were frozen at −80°C.

Postmortem tissue samples were collected and frozen at −80°C. They were thawed and added to 2 mL DMEM supplemented with 2% FBS, 1% antibiotic/antimycotic, and 0.1 vol 10× sucrose phosphate glutamate (SPG). The samples were bead-homogenized (Tissuelyser II, Qiagen) and centrifuged at 14,000 rpm for 10 min. The supernatants were stored at −80°C.

### Immunohistochemistry (IHC)

Four-micrometer sections on charged slides were routinely deparaffinized, followed by heat-induced epitope retrieval using a citrate buffer (Antigen Retrieval Citra Solution pH 6, BioGenex HK086-9K) for 15 min at 110, 3% H_2_O_2_ for 20 min, and a blocking solution (Universal Blocking Reagent, BioGenex HK085-5K) for 15 min. Samples were incubated with polyclonal rabbit SARS-CoV-2 nucleocapsid antibody (Invitrogen PA-41098) diluted 1:100 for 60 min, incubated with biotinylated goat-anti rabbit diluted 1:100 (Vector BA-1000) for 10 min, followed by streptavidin labeled with alkaline phosphatase (+4 Streptavidin AP label, BioCare Medical AP605H) for 10 min, fast red chromogen (Warp red, BioCare Medical) for 5 min, and stained with hematoxylin and eosin (H&E).

### Enzyme-linked immunosorbent assay (ELISA)

Serum IgG ELISA was performed as previously described (13). Briefly, Immulon 2HB 96-well microtiter plates were coated with SARS-CoV-2 S protein from the original WA1 strain at 1 µg/mL (13). The AGM sera were serially diluted 3-fold in blotto (KPL wash buffer +5% nonfat milk +0.5% bovine serum albumin), and 100 µL was incubated on the plates for 2 h. After washing, the plates were incubated with horseradish peroxidase (HRP)-labeled goat anti-monkey IgG secondary antibody (Southern Biotech, Birmingham, Alabama) and developed with KPL SureBlue Reserve TMB Microwell Peroxidase Substrate (SeraCare Life Sciences, Inc., Milford, Massachusetts). Antibody titers were calculated as log_10_ of the highest serum dilution at which the OD_450_ was greater than two standard deviations above the mean OD_450_ of naive serum.

Serum and nasal wash IgA ELISAs were performed as described above, except for the secondary antibody, HRP-conjugated mouse anti-monkey IgA antibody (BioRad catalog no. MCA2553, Hercules, California). For bronchoalveolar lavage (BAL) ELISA, 2-fold diluted sample was incubated on S protein-coated plates (Sino Biological US Inc. catalog no. 40589-V08H4, Wayne, Pennsylvania) for 1 h followed by incubation with mouse anti-monkey IgA secondary antibody. Antibody titers were calculated as log_10_ of the highest serum dilution at which the OD_450_ was greater than 0.2.

### Microneutralization assays

To quantify humoral SARS-CoV-2-neutralizing antibodies, microneutralization assays were performed in a BSL-3 facility. AGM sera were heat-inactivated at 56°C for 45 min and serially diluted 2-fold. The sera were mixed 1:1 with 6 × 10^3^ focus-forming units (FFU)/mL SARS-CoV-2 Wuhan strain and incubated at 37°C for 1 h before being added to 96-wells of Vero cells. One hour post-infection, a methylcellulose overlay (DMEM + 5% fetal bovine serum + 1% P/S + 1% methylcellulose) was added on top of the serum/virus mixture, and the plates were incubated at 37°C, 5% CO_2_ for 24 h. The cells were fixed with 60% methanol/40% acetone and immunostained with anti-SARS-CoV-2 N mouse monoclonal antibody (ProSci catalog no. 35–579), followed by HRP-conjugated goat anti-mouse IgG (ImmunoReagents Inc., Raleigh, North Carolina, USA), and developed with an AEC Substrate Kit (Vector Laboratories, Newark, California, USA). Positively stained cells were counted with a Cytation 7 imaging reader (BioTek). Neutralizing antibody titers are reported as the highest serum dilution at which the virus infectivity was reduced by at least 50%.

### Intracellular cytokine staining (ICS) and flow cytometry

Peripheral blood mononuclear cells (PBMCs) were prepared from AGM blood and cryopreserved. The PBMCs were thawed, and 2 × 10^6^ cells were stimulated with a SARS-CoV-2 WA1 S peptide pool (BEI Resources catalog #NR-52402) at a final concentration of 1 µg/mL in the presence of 1 µg/mL anti-CD28 ECD (Beckman Coulter clone CD28.2), anti-CD107a FITC (eBioscience clone eBioH4A3), and anti-CD49d (BD Biosciences clone 9F10). The PBMCs were stimulated with dimethyl sulfoxide or PMA/ionomycin for negative and positive controls, respectively. After 2 h of incubation at 37°C, 10 µg/mL brefeldin A (BD Biosciences) was added, and the cells were incubated for another 4 h. The cells were washed with PBS and incubated with Aqua-Viability dye (Invitrogen) at room temperature for 15 min. The cells were washed with PBS supplemented with 2% fetal bovine serum (FBS) and incubated at 4°C for 30 min with anti-CD3 Alexa700 (BD Biosciences clone SP34-2), anti-CD4 BV605 (BD Biosciences clone L200), anti-CD8 BV450 (BD Biosciences clone RPA-T8), and anti-CD95 PE-Cy5 (BD Biosciences clone DX2). The cells were washed with PBS + 2% FBS, fixed with Cytofix/Cytoperm (BD Biosciences), permeabilized with Perm/Wash (BD Biosciences), and incubated with anti-IFN-γ PE-Cy7 (BD Biosciences clone B27), anti-TNF-α APC-Cy7 (BioLegend clone Mab11), anti-IL-13 PE (Miltenyi Biotec clone JES10-5A2.2), and anti-MIP-1β APC (eBioscience clone FL34Z3L) antibodies at 4°C for 30 min. The cells were washed with Perm/Wash and PBS + 2% FBS, then resuspended in PBS supplemented with 2% formaldehyde for acquisition on a BD FACSAria Fusion cell sorter. CD3^+^ cells were gated for CD4^+^ and CD8^+^ T cells and separated into memory and naive cells with CD28 and CD95. The net percent of cytokine-secreting cells was determined by subtraction of the values obtained with DMSO-stimulated samples. Data were analyzed using FlowJo software (Version 10).

### IFN-γ enzyme-linked immunospot (ELISpot) assay

ELISpot plates were coated with anti-IFN-γ at 5 µg/mL. The following day, the plates were washed and incubated with complete tumor medium (CTM) S-MEM (Gibco) +10% FBS + 8.6% tumor cocktail (S-MEM + 10% Penicillin/Streptomycin + 7.5% essential amino acids [Gibco] + 0.85% sodium bicarbonate [Sigma-Aldrich] +0.75% dextrose [Sigma-Aldrich] + 0.14% non-essential amino acids [Gibco] + 0.1% sodium pyruvate [Gibco] + 0.1% Glutamax [Gibco] + 0.05% Gentamycin [Sigma-Aldrich]). PBMCs were isolated from blood using Ficoll, and 2 × 10^5^ cells/well were stimulated with a SARS-CoV-2 S peptide pool at 1 µg/mL. Cells were stimulated with media or PMA/Ionomycin for negative and positive controls, respectively. After incubating at 37°C for 20 to 24 h, the plates were washed and sequentially incubated with biotin-conjugated anti-IFN-γ antibody diluted 1:1,000 in PBS supplemented with 0.1% Tween (PBST) and 1% FBS, HRP-conjugated Avidin D diluted 1:1,000, and AEC substrate. The number of IFN-γ-secreting cells was quantified with a CTL-6 ImmunoSpot reader.

### Focus-forming unit (FFU) assay

FFU assays were performed as previously described (13). Briefly, AGM samples were serially diluted in DMEM + 2% FBS + 1% antibiotic/antimycotic and incubated on 96-well plates of Vero E6 cells for 1 h at 37°C, 5% CO_2_, overlayed with medium containing 0.8% methylcellulose, and incubated at 37°C for 20 to 24 h. The cells were immunostained for SARS-CoV-2 infected cells as described earlier, and the number of positively stained cells was reported as FFU/mL.

### Plaque assay

To quantify CVXGA1 in the nasal cavities of vaccinated AGMs, plaque assays were performed. AGM samples were serially diluted in DMEM + 5% FBS + 1% P/S and incubated on Vero cells. The infected cells were overlayed with 2% low melting point agarose in DMEM. Seven days later, the cells were fixed with 2% formaldehyde and stained with crystal violet.

### RT-qPCR

SARS-CoV-2 viral RNA levels were quantified by RT-qPCR as previously described (13).

### Statistics

**Fig 1:**
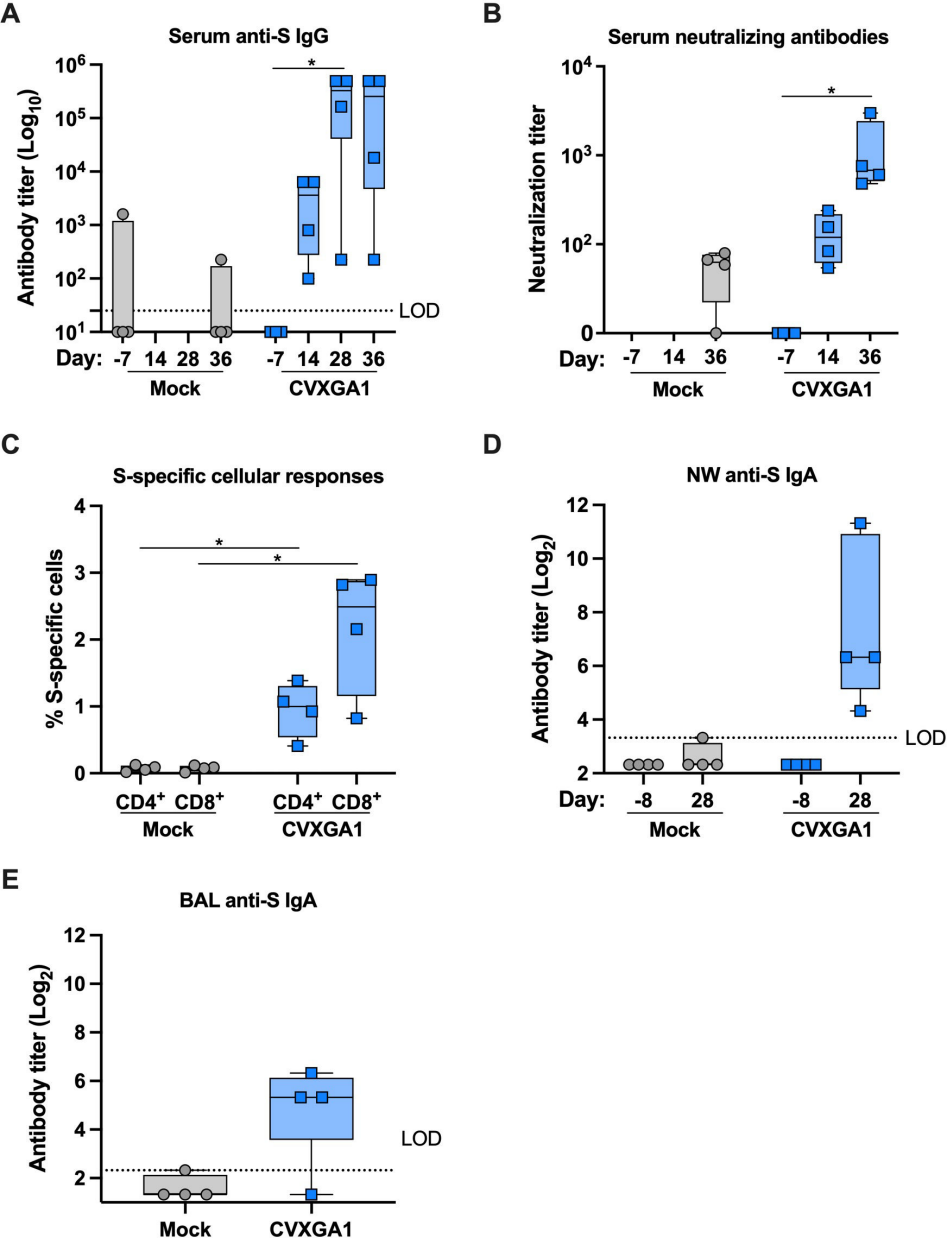
Intranasal immunization of AGMs with CVXGA1 induces neutralizing antibody, mucosal antibody, and cellular responses against SARS-CoV-2. For AGM study 1, AGMs received an intranasal dose of 10^6^ PFU CVXGA1, and unvaccinated monkeys (mock) were used as controls (*n* = 4). Serum was collected on days −7, 14, 28, and 36 post-vaccination. (**A**) Anti-SARS-CoV-2-S IgG antibodies were quantified by ELISA. The lower limit of detection (LOD) is indicated by the dotted line. Statistical significance was calculated for each vaccine group by paired, nonparametric test with Dunn’s multiple comparisons in comparison to day −7 (*P ≤ 0.05). (**B**) Neutralizing antibody titers against SARS-CoV-2 WA1 were calculated as log_10_ of the highest serum dilution at which the virus infectivity was reduced by at least 50%. Statistical significance was calculated for each vaccine group by paired, nonparametric test with Dunn’s multiple comparisons in comparison to immunization at day −7 (*P ≤ 0.05). (**C**) S-specific CD4^+^ or CD8^+^ cells at 14 days post-immunization. The number of S-specific cells is represented as the percent of CD4^+^ or CD8^+^ T cells secreting IFN-γ, TNF-⍺, MIP-β, IL-13, or CD107⍺ relative to all CD4^+^ and CD8^+^ T cells. Statistical significance was calculated for CVXGA1 CD4^+^ and CD8^+^ cellular responses with Mann-Whitney tests compared with the mock group (*P ≤ 0.05). (**D**) Anti-SARS-CoV-2-S IgA antibodies in nasal wash were quantified by ELISA. The LOD is indicated by the dotted line. Statistical significance was calculated for each vaccine group by paired t tests in comparison to prior to vaccination at day −7. (**E**) Anti-SARS-CoV-2-S IgA antibodies in bronchioalveolar were quantified by ELISA. The LOD is indicated by the dotted line. Statistical significance was calculated with a Mann-Whitney test.

**Fig 2:**
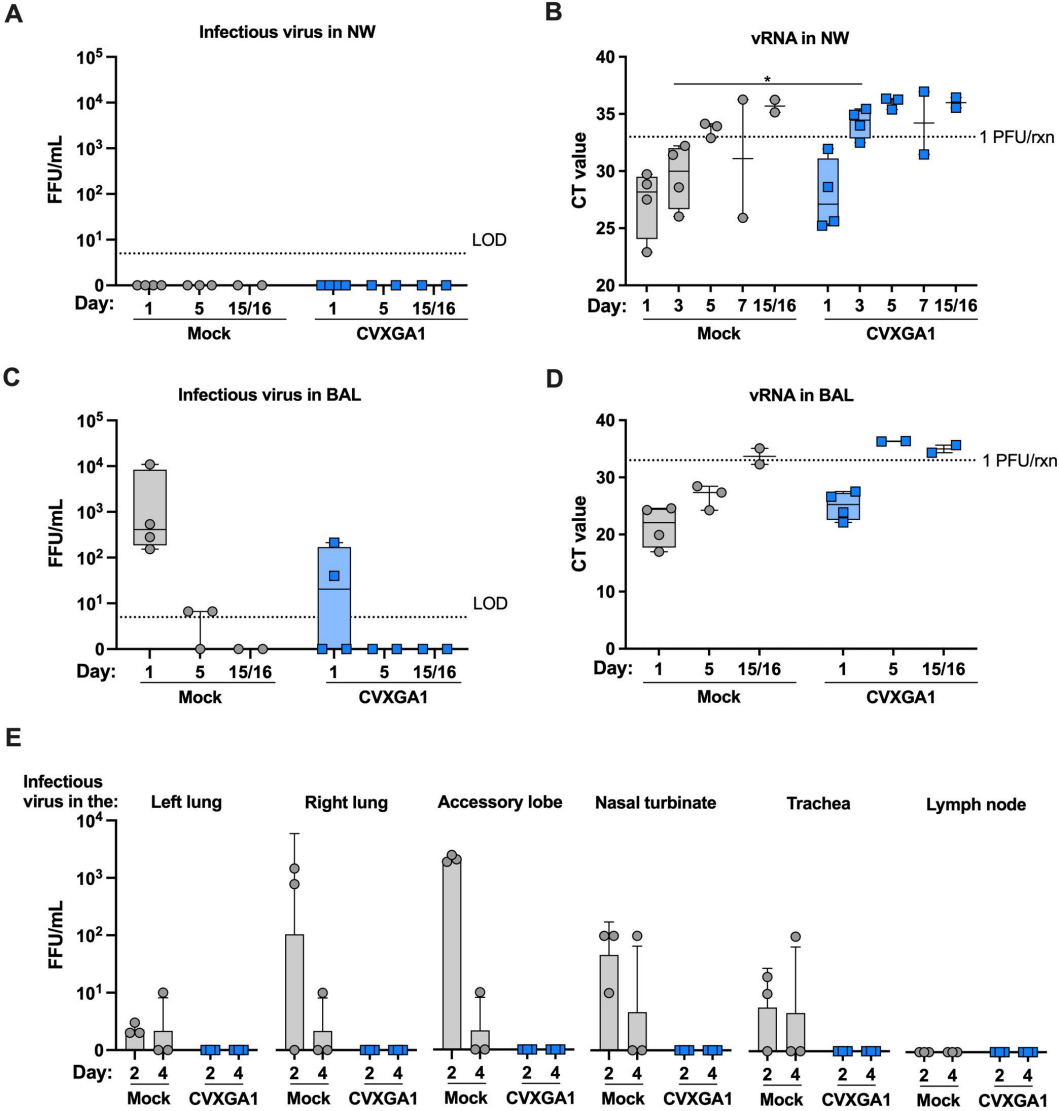
Intranasal immunization with CVXGA1 protects AGMs from challenge with SARS-CoV-2. Viral loads in AGM nasal wash (NW; **A**) and bronchial lavage (BAL; **C**) were quantified by focus-forming unit (FFU) assay in Vero E6 cells and graphed as FFU/mL for AGM study 1. The LOD is indicated by the dotted line. vRNA load in NW (**B**) and BAL (**D**) were quantified with RT-qPCR. The cycle threshold (Ct) value for each sample is presented. For days 1, 3, 5, 7, and 15/16, the values for CVXGA1-vaccinated animals are compared with the values for unvaccinated animals (mock) with nonparametric Mann-Whitney tests (*P ≤ 0.05). (**E**) Viral loads in AGM tissues were quantified by FFU assay and expressed as FFU/mL in AGM study 2. AGMs were intranasally immunized with 10^6^ PFU CVXGA1 (*n* = 4) or mock vaccinated AGMs (*n* = 4). The AGMs were challenged with 5 × 10^5^ PFU per nostril SARS-CoV-2 alpha variant at day 28. At 2 and 4 days post-challenge, one AGM from each vaccination group was euthanized, tissues were collected, and infectious virus was quantified via focus-forming unit (FFU) assays in triplicate. For days 2 and 4, the values for CVXGA1-vaccinated animals were compared with the values for unvaccinated animals with nonparametric Mann-Whitney tests.

Sample sizes were chosen to provide useful data while being ethically responsible. All statistics were calculated using Prism version 9.3.1 (GraphPad Software, LLC.), and multiple testing was not accounted for. For IgG ELISA, neutralization, ELISpot, and ICS experiments (Fig. 1A and B, 2A and B, and 7), statistical significance was calculated by comparing all time points with pre-immunization titer by paired, nonparametric test with Dunn’s multiple comparisons. For BAL IgA ELISA (Fig. 1E), statistical significance was calculated with a Mann-Whitney test. For the PBMC ICS experiments (Fig. 1C and 6A and B), CVXGA1-vaccinated cellular responses were compared with unvaccinated cellular responses with nonparametric Mann-Whitney tests. For the nasal wash IgA ELISA (Fig. 1D), day 28 values were compared with day −8 values for each vaccine group using a paired t test. For the infectious virus FFU (Fig. 2A, C, and E) and vRNA RT-qPCR (Fig. 2B and D) experiments, values for unvaccinated animals were compared with values for CVXGA1-vaccinated animals with nonparametric Mann-Whitney tests. Significance is indicated as follows: *P ≤ 0.05, **P < 0.01.

## RESULTS

### Intranasal immunization with CVXGA1 induces robust immune responses in AGMs

**Fig 3:**
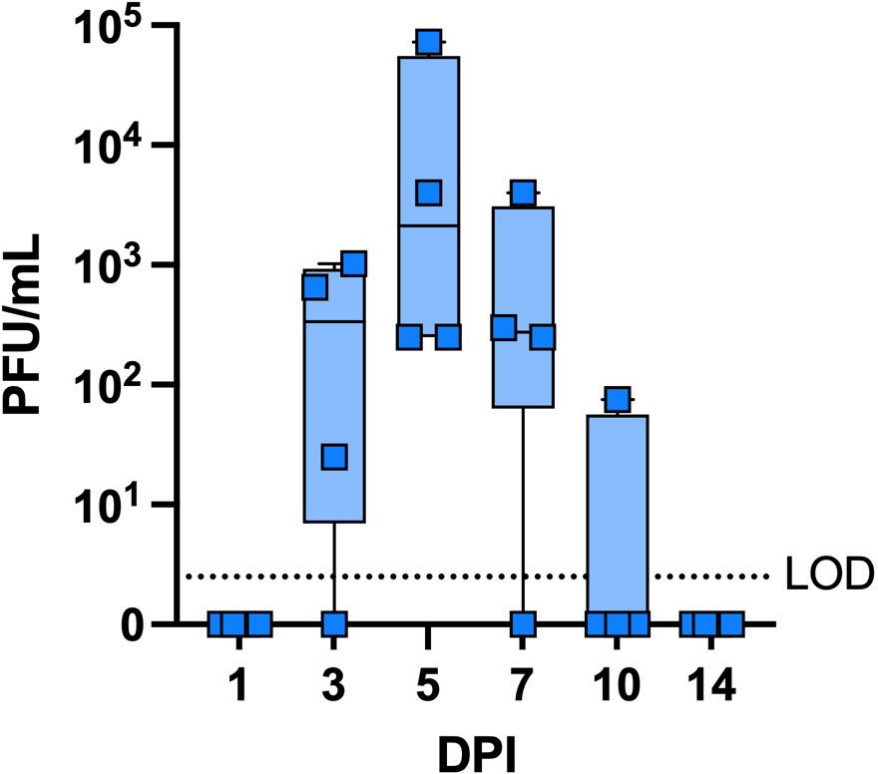
CVXGA1 shedding in the nasal cavity of vaccinated AGMs. Plaque assays were performed with AGM nasal wash samples to quantify CVXGA1 found in the nasal cavities of vaccinated AGMs. The limit of detection (LOD) is indicated by the dotted line.

The immunogenicity and protective efficacy of CVXGA1 have been reported for mice, ferrets (13), and hamsters. To assess its immunogenicity in a nonhuman primate model, AGMs were administered a single, intranasal dose of 10^6^ plaque-forming units (PFU) CVXGA1 for AGM study 1. Nasal wash samples were collected to monitor vaccine shedding from the nasal cavities of vaccinated AGMs. Peak viral shedding was detected at day 5 with a mean titer of over 10^3^ PFU/mL. No CVXGA1 was detected in the nasal wash samples of AGMs by day 14 post-immunization (Fig. 3). Blood was collected at days −7, 14, 28, and 36 post-immunization, and anti-SARS-CoV-2-S IgG antibodies were measured via ELISA. Unvaccinated or mock-immunized AGMs were used as negative controls, and surprisingly, one AGM had anti-S antibodies before study commencement. For AGMs vaccinated with CVXGA1, mean S IgG antibody titers were greater than 5 log_10_ at days 28 and 36 post-immunization (Fig. 1A). A neutralization assay with SARS-CoV-2 WA1 strain revealed that CVXGA1 generated mean neutralizing antibody titers over 2 log_10_ at 36 days post-immunization (Fig. 1B). The S-specific CD4^+^ and CD8^+^ T-cell responses in isolated PBMCs were quantified with intracellular cytokine staining (ICS) for IFN-γ, TNF-⍺, MIP-β, IL-13, and CD107⍺, and positive S-specific CD4^+^ and CD8^+^ T cells were quantified (Fig. S2). Compared with unvaccinated AGMs, AGMs vaccinated with CVXGA1 had a significant increase in total CD4^+^ and CD8^+^ T-cell responses with median 1.0 and 2.5 percent S-specific cells, respectively (Fig. 1C), most of which secreted IFN-γ, TNF-⍺, or CD107⍺, and not MIP-β or IL-13. To measure the mucosal antibody response induced by CVXGA1, anti-SARS-CoV-2-S IgA antibodies in nasal swabs collected prior to vaccination (day −8) and 28 post-immunization, as well as BAL samples collected on day 15 post-immunization, were quantified via ELISA. All four CVXGA1-vaccinated AGMs generated S-specific IgA antibodies in their upper respiratory tract at 28 days post-immunization (Fig. 1D), and three of four had detectable levels of S-specific IgA antibodies in their lower respiratory tract 15 days post-immunization (Fig. 1E). These data demonstrate that intranasal immunization with CVXGA1 induces mucosal immune response.

### Intranasal immunization with CVXGA1 protects African green monkeys against SARS-CoV-2 challenge

To test the protective efficacy of CVXGA1 in AGMs, the AGMs from AGM study 1 were challenged intranasally with SARS-CoV-2 at 36 days post-immunization. Although no infectious SARS-CoV-2 was detected in any nasal wash samples (Fig. 2A), unvaccinated AGMs had median CT values of 28.2 and 30.0, indicative of infectious virus, on days 1 and 3 post-challenge in their nasal cavities, respectively (Fig. 2B). Compared with the unvaccinated AGMs, CVXGA1-vaccinated AGMs had statistically significantly less vRNA in their nasal cavities at day 3 with all but one vaccinated AGM having virus-negative CT values. On day 1 post-challenge, all unvaccinated AGMs had infectious SARS-CoV-2 in their BAL with a median of 2.6 log_10_ FFU/mL, whereas only two of four CVXGA1-vaccinated AGMs had detectable virus with median titer of more than 10-fold lower than the control AGMs (Fig. 2C). vRNA levels in BAL were consistent with the infectious virus results. Unvaccinated AGMs had detectable viral RNA above 1 PFU/mL detected on days 1 and 5, but CVXGA1-immunized AGMs cleared all vRNA after day 1 (Fig. 2D).

The second AGM (AGM study 2) study was to assess the tissue viral burden at 2 and 4 days following challenge after immunization with an intranasal dose of 10^6^ PFU CVXGA1. Four unvaccinated, or mock, AGMs were used as controls. At 2 days post-challenge, the unvaccinated AGM had a mean infectious virus of 0.3, 2.0, 3.3, 1.7, 0.8 log_10_ FFU/mL in their left lung, right lung, accessory lobe, nasal turbinate, and tracheas, respectively. At 4 days post-challenge, infectious virus was still detected in all tissues other than the lymph node. In contrast, none of the CVXGA1-immunized AGMs had a detectable virus in any of their tissues at either time point (Fig. 2E).

**Fig 4:**
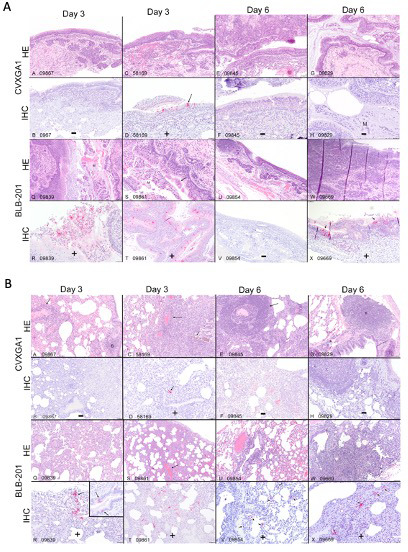
Intranasal vaccination of AGMs with CVXGA1 reduces SARS-CoV-2 pathology and viral burden in the nasal turbinates and lungs. AGMs in study 3A received a single intranasal dose of 10^6^ PFU BLB-201 or CVXGA1. At 40 days post-immunization, the AGMs were challenged with SARS-CoV-2 alpha variant. At 3 and 6 days post-challenge, two AGMs per vaccine group were euthanized. Nasal turbinate (**A**) and lung tissues (**B**) were collected and processed for hematoxylin and eosin (HE) staining (top) and SARS-CoV-2 immunohistochemistry (IHC, bottom). Negative and positive IHC for SARS-CoV-2 is indicated by – or +, respectively.

A third AGM (AGM study 3) study was divided into two sub-studies. The purpose of study 3A was to assess the efficacy of CVXGA1 at reducing disease pathology and SARS-CoV-2 viral burden in the upper and lower respiratory tracts following heterologous challenge with the alpha variant. Following a single intranasal dose of 10^6^ PFU of a control virus (BLB-201, PIV5 encoding RSV F gene) or CVXGA1, AGMs were challenged with the alpha variant. At days 3 and 6 post-challenge, nasal turbinate and lung tissues were collected and processed for H&E staining and SARS-CoV-2 immunohistochemistry (IHC). Lymphocyte infiltration was observed in all nasal turbinate samples with the most severe rhinitis and inflammation seen in one CVXGA1-immunized AGM on day 3 (subpanel C) and three of four BLB-201-immunized AGMs on days 3 and 6 (subpanels Q, S, and W) (Fig. 4A). Nasal turbinate H&E images were scored on a scale of 0 (none) to 3 (severe) for rhinitis. Rhinitis scores for CVXGA1-immunized AGMs were 1 and 3 for days 3 and 2 and 1 for day 6, whereas scores for BLB-201-immunized AGMs were 3 and 2 for days 3 and 1 and 3 for day 6 (Table 1). Inflammation in nasal turbinate H&E slides correlated with positive SARS-CoV-2 IHC with one of four CVXGA1-immunized AGMs and three of four BLB-201-immunized AGMs having positive staining (Fig. 4A). Varying levels of pneumonia and bronchitis were evident in all AGM lung tissues (Fig. 4B). Sections from all seven lung lobes were scored for inflammation, hyperplasia, and alveolar exudate for a total maximum combined score of 98 being the most severe. Total lung scores for CVXGA1-immunized AGMs ranged from 5 to 22, whereas scores for BLB-201-immunized AGMs ranged from 18 to 35 (Table 1). IHC of lung tissues revealed that although only one of four CVXGA1-immunized AGM lungs stained positive, lungs from all four BLB-201-immunized AGMs stained positive for SARS-CoV-2 (Fig. 4B). Together, these data show that CVXGA1 protects against challenge with SARS-CoV-2 Wuhan and alpha variant and reduces lung disease pathology.

**TABLE 1:** Summary of pathological changes

Animal no.	Rhinitis (inflammation (0–3) [IHC]^*a*^	Tracheitis (inflammation 0–3)^*b*^	Bronchitis (0–4/lobe; 28 total)	Pneumonia extent score (0–4/lobe; 28 total)^*c*^	Total lung score (0–98)^*d*^	Syncytia (yes/no) [hyperplasia]^*e*^	Perivasculitis (0–3/lobe; 21 total)	BALT (0–3/lobe; 21 total)	IHC (1–2 slides)-lung^*f*^
CVXGA1									
09867	1 [Neg]	2	8	2	5	No [2]	2	12	Neg
58169	3 [Pos]	1	8	4	10	No [3]	3	5	Pos
09845	2 [Neg]	2	11	9	22	No [5]	6	0	Neg
09829	1 [Neg]	1	7	6	15	No [1]	0	8	Neg
BLB-201									
09839	3 [Pos]	1	2	11	26	No [3]	2	1	Pos
09861	2 [Pos]	2	14	13	35	No [8]	4	7	Pos
09854	1 [Neg]	1	11	6	18	No [3]	5	3	Pos
09669	3 [Pos]	2	7	6	20	Yes [4]	6	4	Pos

^*a*^ Immunohistochemistry results for Sars-CoV-2 are in brackets and given as Neg = no staining and Pos = immunopositivity.

^*b*^ 0 = none/minimal; 1 = mild; 2 = moderate; 3 = severe.

^*c*^ Extent score was based on percentage involvement, and a 0–4 score (0 = none; 1 = 10%; 2 = 10%–25%; 3 = 25%–75%; 4 = 75%) for each lung lobe was summed for a possible total of 28.

^*d*^ Based on four parameters per lobe (extent 0–4; bronchiolar/septal inflammation 0–3; bronchiolar/alveolar hyperplasia 0–3; and alveolar exudate 0–4) summed for a possible score of 98.

^*e*^ Syncytia were scored whether present. Hyperplasia of bronchiolar/alveolar septal epithelium is in parentheses and was scored 0 = none, 1 = mild, 2 = moderate, and 3 = severe per lobe for a possible total score of 21.

^*f*^ IHC = immunohistochemistry for SAR-CoV-2, which was scored as Neg = no staining or Pos = immunopositive staining.

### Intranasal immunization with CVXGA1 induces a long-lasting humoral response in AGMs

**Fig 5:**
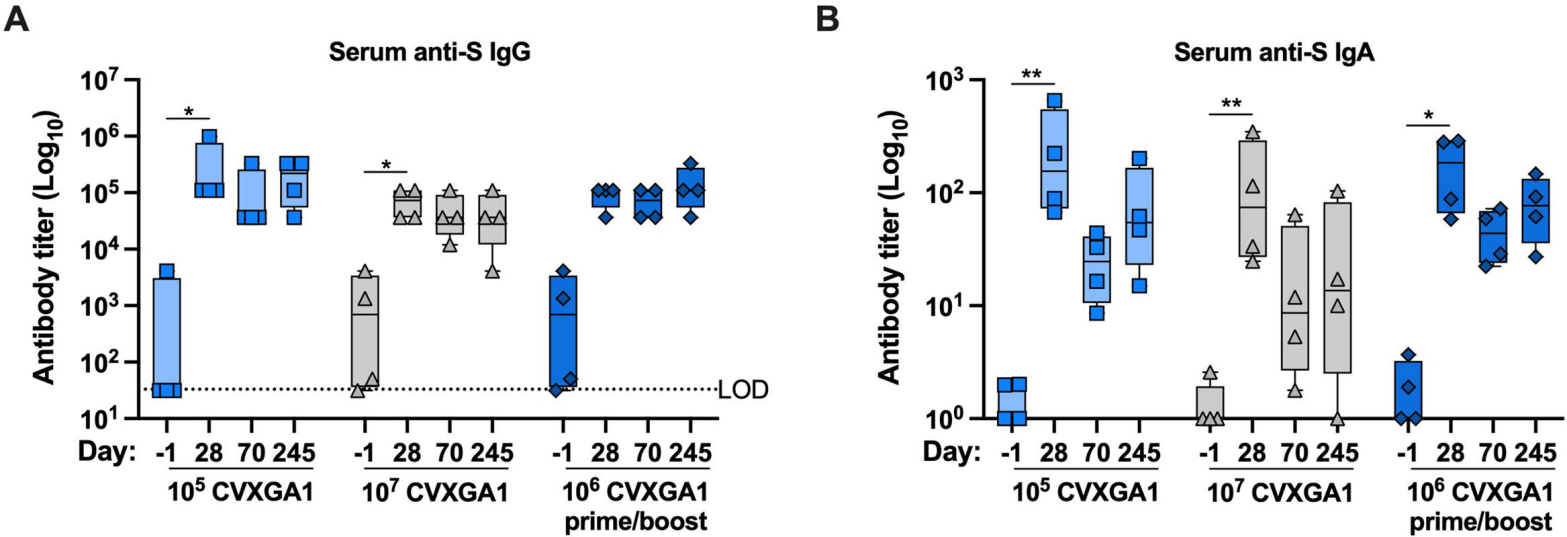
Intranasal vaccination of AGMs with CVXGA1 induces a long-lasting humoral response. AGMs in study 3B received a single intranasal dose of 10^6^ PIV5 BLB-201, 10^5^ PFU CVXGA1, 10^7^ PFU CVXGA1, or two doses of 10^6^ PFU CVXGA1 at 28 days intervals. (**A**) Anti-SARS-CoV-2-S IgG antibody titers were quantified by ELISA. The LOD is indicated by the dotted line. Statistical significance was calculated for each vaccine group by paired, nonparametric ANOVA with Dunn’s multiple comparisons in comparison to day −7 (*P ≤ 0.05). (**B**) Serum anti-SARS-CoV-2-S IgA antibodies were also quantified by ELISA. The LOD is indicated by the dotted line. Statistical significance was calculated for each vaccine group by paired, nonparametric test with Dunn’s multiple comparisons in comparison to day −7 (*P ≤ 0.05, **P < 0.01).

To assess the longevity of CVXGA1-induced immunity, the second sub-study of AGM study 3, 3B, was conducted. AGMs (*n* = 4) received an intranasal dose of 10^5^, 10^6^, or 10^7^ PFU CVXGA1, and the AGMs who received 10^6^ PFU received a second dose (prime/boost) of 10^6^ PFU CVXGA1 at day 28. Five AGMs that had pre-existing anti-SARS-CoV-2 antibodies were included to assess vaccine-boosting efficacy. Blood was collected at days −1, 28, 70, and 245 post-immunization, and serum anti-SARS-CoV-2-S IgG and IgA antibodies were quantified via ELISA. At 28 days post-immunization, all AGMs had high levels of anti-SARS-CoV-2 IgG antibodies with median titers of 5.0, 5.0, and 4.8 log_10_ for doses 10^5^, 10^6^, and 10^7^ PFU CVXGA1, respectively. Additionally, all AGMs who were SARS-CoV-2 serum positive before immunization (*n* = 1, 10^5^ CVXGA1; *n* = 2, 10^7^ CVXGA1; *n* = 2, 10^6^ CVXGA1) had at least a 10-fold increase in their antibody titers at day 28 (Fig. S1). All three vaccinated AGM groups maintained their antibody titers for 245 days with no statistical significance between days 28, 70, and 245 (Fig. 5A).

Serum IgA antibody titers were assessed in a similar manner. At 28 days post-immunization, all AGMs had high levels of anti-SARS-CoV-2 IgA antibodies with median titers of 2.1, 2.1, and 1.9 log_10_ for doses 10^5^, 10^6^, and 10^7^ PFU CVXGA1, respectively. These values are approximately a 50-fold increase from the IgA antibody titers prior to immunization in each group. The IgA antibody titers fluctuated at day 70 and day 245 post-immunization for all groups, but they were not statistically significant from day 28 post-immunization titers, and the boosting effect was not apparent (Fig. 5B). These results demonstrate that intranasal immunization with CVXGA1 induces long-lasting antibody responses.

### Intranasal immunization with CVXGA1 induces long-lived cellular responses in AGMs

**Fig 6:**
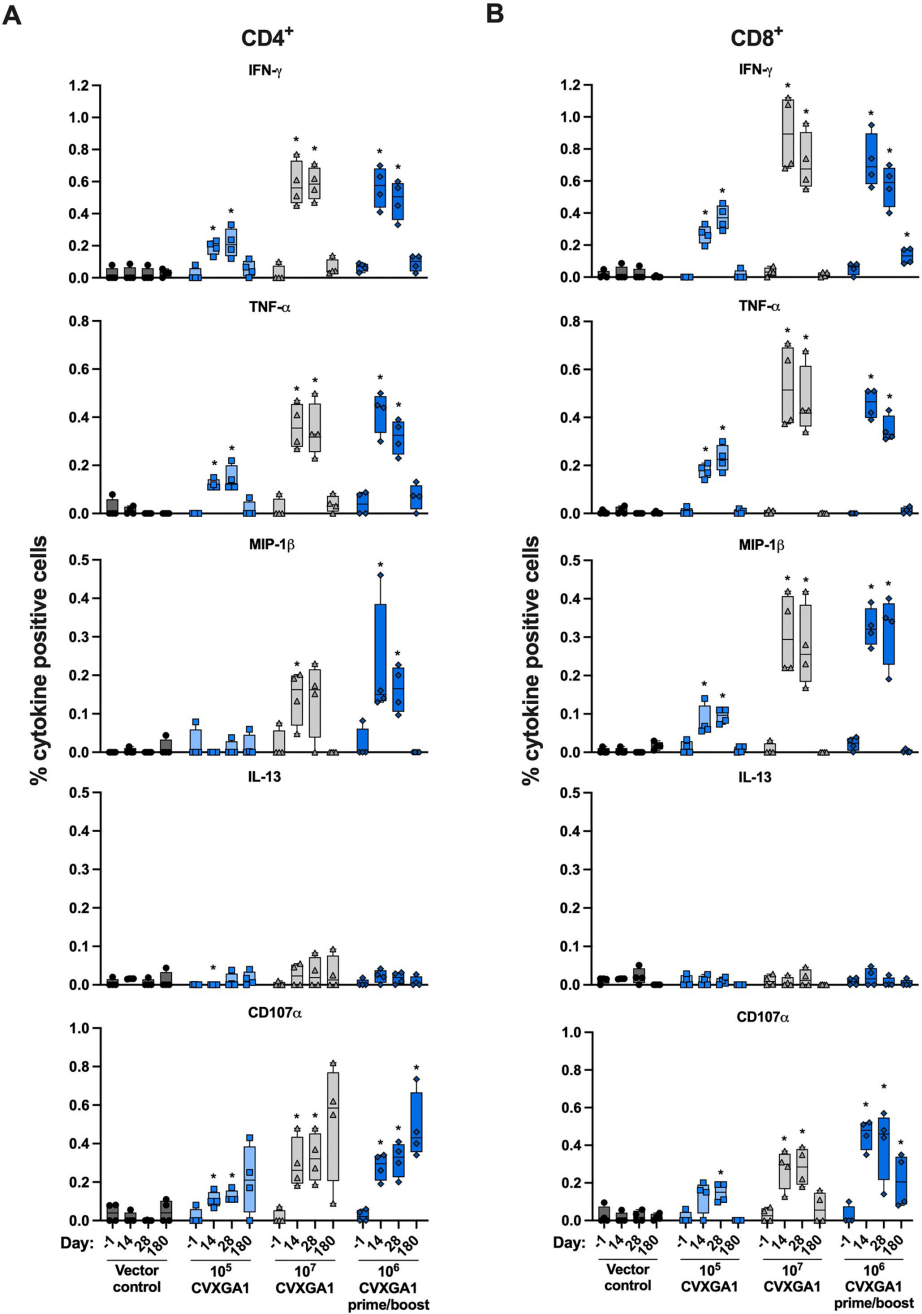
Intranasal vaccination of AGMs with CVXGA1 induces long-lasting CD4^+^ and CD8^+^ T-cell responses. CD4^+^ (**A**) and CD8^+^ (**B**) SARS-CoV-2-specific cellular immune responses sorted by IFN-γ, TNF-α, MIP-1β, IL-13, or CD107α expressing cells. The values for CVXGA1-vaccinated animals are compared with the values for BLB-201-vaccinated animals with nonparametric Mann-Whitney tests (*P ≤ 0.05).

The cellular immune response to SARS-CoV-2 plays a significant role in reducing COVID-19 disease severity (17). To assess the cellular immune response induced by CVXGA1, blood was collected at days −1, 14, 28, and 180 post-immunization. Isolated PBMCs were used to perform intracellular cytokine staining (ICS) to quantify S-specific IFN-γ-, TNF-⍺-, MIP-β-, IL-13-, and CD107⍺-secreting CD4^+^ (Fig. 6A) and CD8^+^ (Fig. 6B) T cells. Overall, CVXGA1-immunized AGMs generated S-specific CD4^+^ and CD8^+^ T cells producing high levels of IFN-γ, TNF-⍺, MIP-β, or CD107⍺ cytokines and very low levels of IL-13, indicative of a type 1 immune response. For all CVXGA1 vaccine groups, IFN-γ- and TNF-⍺-secreting CD4^+^ and CD8^+^ T-cell titers peaked on days 14 or 28 post-immunization. For the 10^7^ PFU dose group, median IFN-γ-secreting CD4^+^ and CD8^+^ T-cell titers reached 0.6 and 0.9 percent, respectively, whereas median TNF-⍺-secreting CD4^+^ and CD8^+^ T-cell titers reached 0.4 and 0.5 percent, respectively. Comparing the responses induced by AGMs who received a single dose of 10^5^ or 10^7^ PFU CVXGA1, AGMs who received the higher dose had more robust CD4^+^ and CD8^+^ T-cell responses. For example, on day 28, AGMs who received 10^7^ PFU CVXGA1 had median levels of IFN-γ-secreting CD4^+^ and CD8^+^ T cells 2.8-fold and 3.3-fold, respectively, higher than AGMs who received 10^5^ PFU CVXGA1. At day 180, AGMs who received 10^7^ PFU CVXGA1 had median levels of CD107⍺-secreting CD4^+^ and CD8^+^ T cells 2.8-fold and 0.1-fold higher than AGMs who received 10^5^ PFU CVXGA1 (Fig. 6A and B).

**Fig 7:**
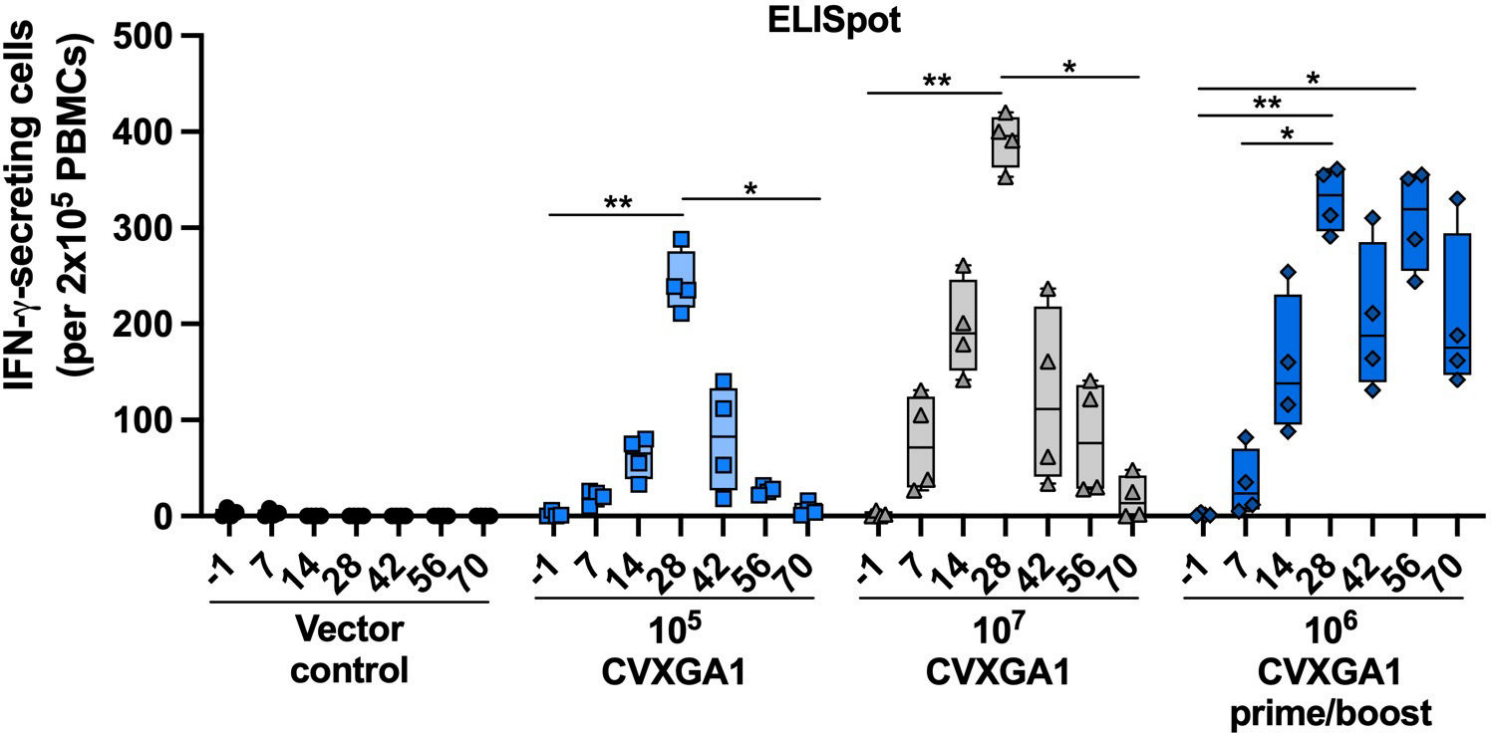
Intranasal vaccination of AGMs with CVXGA1 induces a long-lasting S-specific cellular response. The number of IFN-γ-secreting cells per 2 × 10^5^ PBMCs on different days post-vaccination with BLB-201 or CVXGA1 were quantified in AGM study 3. Statistical significance was calculated for each vaccine group by paired, nonparametric test with Dunn’s multiple comparisons in comparison to vector control (*P ≤ 0.05, **P < 0.01).

ELISpot assay that measures cytotoxic T cells (CTL) was performed with the AGM’s PBMCs collected at days −1, 7, 14, 28, 42, 56, and 70 post-immunization. Following a single dose of CVXGA1, peak numbers of IFN-γ-secreting PBMCs were observed at day 28 post-immunization with the highest response being over 400 IFN-γ-secreting cells per 2 × 10^5^ PBMCs for AGMs that received 10^7^ PFU CVXGA1. Following the boost, AGMs who received two doses of 10^6^ PFU CVXGA1 had a 1.7-fold increase in median IFN-γ-secreting cells from days 42 to 56. In contrast to the AGMs who received one dose of CVXGA1, the IFN-γ-secreting cells in the prime/boost AGMs did not reduce significantly at day 70 when compared with day 28 post-immunization (Fig. 7). Like the humoral immune responses, these results indicate that intranasal immunization with CVXGA1 induces long-lasting cellular immune responses.

## DISCUSSION

Currently approved COVID-19 vaccines reduce COVID-19 disease, hospitalizations, and death. However, immune responses from the COVID-19 mRNA vaccines mainly include serum-neutralizing antibodies that decline rapidly and limit mucosal and cellular immune responses (5, 18, 19). PIV5-vectored CVXGA1 antibody responses and efficacy of intranasal immunization have been evaluated in mice, ferrets, hamsters, and cats. Limited reagents and sample volumes hindered our ability to investigate mucosal and cellular responses from CVXGA1 immunization in small animal models. Thus, we conducted the AGM studies described here to further examine CVXGA1-elicited immune responses, including serum and mucosal antibodies as well as cellular immune responses, in vaccine-mediated protective efficacy.

A mucosal IgA response is critical for protection against SARS-CoV-2 infection and transmission (20), and S RBD-specific salivary IgA antibodies have been found to positively correlate with protection against breakthrough infections of Omicron BA.1 variant (21). Intramuscular immunization of SARS-CoV-2 mRNA vaccines induces a weak mucosal IgA response that can be boosted by natural infection (20). Intranasal vaccination has been shown to elicit local mucosal immunity, as reported for parainfluenza virus 3 (PIV3)-vectored SARS-CoV-2 vaccine in Rhesus macaques (22). Our AGM studies have demonstrated that a single, intranasal dose of CVXGA1 induces a mucosal IgA antibody response in the upper and lower respiratory tracts of AGMs, although levels of IgA antibodies and correlation of protection have yet to be established (Fig. 1D and E).

A robust cellular response, especially IFN-γ-secreting PBMCs, against SARS-CoV-2 is important for protection against SARS-CoV-2 variants (23). In NHPs, intramuscular SARS-CoV-2 mRNA vaccination induces an antigen-specific CD4^+^ T-cell response but is deficient in generating a CD8^+^ T-cell response (18). In contrast, a single, intranasal dose of CVXGA1 induces SARS-CoV-2-S-specific CD4^+^ and CD8^+^ T cells, as well as high levels of IFN-γ-secreting CTL responses (Fig. 1C and 7). Furthermore, the S-specific CD4^+^ and CD8^+^ T cells produced high levels of IFN-γ and TNF-⍺, but very low levels of IL-13, indicative of a type 1 immune response (Fig. 6A and B). SARS-CoV-2 S protein T-cell epitopes are well conserved (23), and the CMI response from CVXGA1 is expected to offer cross-protection against the variants. It has been reported for humoral immunity against influenza that vaccination with an antigenically drifted vaccine strain tends to boost original vaccine-mediated antibodies (24) and that vaccination against COVID-19 provides a broader cross-reactivity of antibodies than natural infection (25). However, this has yet to be determined for SARS-CoV-2 cellular immune responses. Since the CMI responses are more conserved, the strong CMI immunity from intranasal PIV5-vectored COVID-19 vaccine could offer broader protection against emerging variants.

Our challenge model was not as robust as previously published data, due to the lack of infectious challenge virus in the nasal wash of mock immunized AGMs (Fig. 2A). This may be in part due to the fact that we challenged intranasally, whereas other studies used combined intranasal and intratracheal challenge routes (6). However, we were able to detect challenge virus RNA in the upper and lower respiratory tracts, as well as infectious virus in the lower respiratory tracts, to provide some protection results. CVXGA1-immunized AGMs cleared challenge virus vRNA from their upper and lower respiratory tracts (Fig. 2B and D) and infectious challenge virus from their lower respiratory tracts (Fig. 2C) faster than unvaccinated AGMs (Fig. 2C). Importantly, no infectious SARS-CoV-2 was detected in the tissues of CVXGA1-immunized AGMs (Fig. 2E). Following challenge with SARS-CoV-2 alpha variant, only one of two CVXGA1-vaccinated AGMs had positive IHC staining for SARS-CoV-2 in their nasal turbinate at day 3 and neither AGM had positive staining at day 6 post-challenge (Fig. 4A). The rapid clearance of SARS-CoV-2 vRNA in the upper respiratory tract of AGMs (Fig. 2B) should reduce viral transmission, and these data demonstrate that intranasal immunization of CVXGA1 reduces and shortens the duration of viral burden following intranasal challenge infection in AGMs.

During the duration of our study in AGMs, CVXGA1-induced anti-S IgG antibody titers remained consistent (Fig. 5A) and anti-S IgA serum antibody titers showed a minimal decline (Fig. 5B). Although we did not examine cross-reactivity of CVXGA1-induced immune responses in AGMs, we found that in hamsters previously immunized with a COVID-19 mRNA vaccine, intranasal immunization with CVXGA1 boosts neutralizing antibody titers against WA1, delta, and omicron variants (26). For CVXGA1-induced cellular responses, we were able to detect IFN-γ- and TNF-⍺-secreting CD4^+^ T cells at day 245 (Fig. 6A) and an increase in S-specific CD107⍺-secreting CD4^+^ T cells at day 180 post-immunization (Fig. 5A). This CD4^+^ T cell response may have played a role in the long-lived antibody responses observed at day 245 post-immunization (Fig. 5A), as has been observed in convalescent human subjects (27). Furthermore, CVXGA1 prime/boosted AGMs had IFN-γ-secreting T cell levels over 150 IFN-γ-secreting cells per 2 × 10^5^ PBMCs, similar to their levels at day 42 (Fig. 7), indicating that a second dose enhanced the longevity of the cellular response.

For AGM study 1, several unvaccinated AGMs developed neutralizing antibodies (Fig. 1B). It is possible that the animals were exposed to either SARS-CoV-2 or CVXGA1-immunized animals. Prior to beginning AGM study 3, we found that several AGMs had pre-existing anti-SARS-CoV-2-S antibodies, probably due to exposure to animal handlers, and were included in the longitudinal study. All AGMs with pre-existing anti-SARS-CoV-2-S antibodies had antibody titers increase following CVXGA1 immunization (Fig. S1; Fig. 5A) and generated S-specific IFN-γ-secreting PBMCs (Fig. 6), as well as IFN-γ- and TNF-⍺-secreting CD4^+^ and CD8^+^ T cells (Fig. 6A and B). These data indicate that CVXGA1 can boost immune responses in individuals with pre-existing immunity to SARS-CoV-2. Pre-existing vector-specific immunity is also of concern for viral-vectored vaccines. Although pre-existing immunity against PIV5 did not inhibit the immunogenicity of a PIV5-vectored vaccine in dogs (28), high levels of anti-PIV5 antibodies may affect immunogenicity of a PIV5-vectored vaccine in NHPs (29). In this study, we found that a second dose of CVXGA1 enhanced the longevity of the induced serum IgA antibody (Fig. 5B) and cellular responses (Fig. 6 and 7). Because approximately 30% of the human population has detectable anti-PIV5 antibodies based on a previous study (28), the effect of pre-existing PIV5 immunity on vaccine-mediated antigen-specific responses is being examined in our PIV5-vectored RSV and COVID-19 vaccine clinical trials (15, 30).

The AGM data presented here shows that a single, intranasal dose of CVXGA1 elicits mucosal, humoral, and cellular immune responses to SARS-CoV-2 and offers protection to AGMs from challenge with SARS-CoV-2. CVXGA1-generated immunity is long-lasting and is likely to enhance pre-existing immunity in convalescent and already-immunized individuals. This intranasal vaccine may help reduce the transmission of SARS-CoV-2 virus between individuals and potentially decrease the emergence of new variants. Finally, the CVXGA1 spike gene may be updated to enhance variant-specific immunity and protect against future variants of concern.

## Data Availability

All data needed to evaluate the conclusions in the paper are present in the paper and/or the supplemental material. Requests for the SARS-CoV-2 and CVXGA1 viruses should be submitted to the University of Georgia Research Foundation pending scientific review and a completed material transfer agreement. Additional data related to this paper may be requested from the authors.

## SUPPLEMENTAL MATERIAL

Supplemental figures jvi.01990-24-s0001.pdf Figures S1 and S2.

ASM does not own the copyrights to Supplemental Material that may be linked to, or accessed through, an article. The authors have granted ASM a non-exclusive, world-wide license to publish the Supplemental Material files. Please contact the corresponding author directly for reuse.
