# Supplementary material for: A parainfluenza virus 5 (PIV5)-vectored intranasal SARS-CoV-2 vaccine (CVXGA1) elicits protective and long-lasting immunity in nonhuman primates
Source: J Virol. 2025 Mar 21;99(4):e01990-24. doi: 10.1128/jvi.01990-24 (PMC11998508; doi:10.1128/jvi.01990-24)
Supplement: Supplemental figures — Figures S1 and S2. [file jvi.01990-24-s0001.pdf]

Supplemental Figures

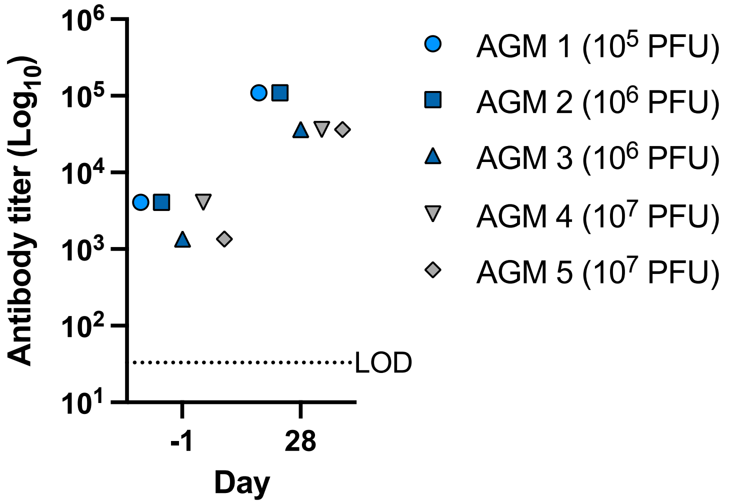

Supplemental figure 1. CVXGA1 boosts the serum IgG antibody response of AGMs with pre-existing anti-SARS-CoV-2-S antibodies.

AGMs in study 3B received a single intranasal dose of 10^5 PFU CVXGA1, 10^6 PFU CVXGA1, or 10^7 PFU CVXGA1. Serum was collected at days -1 and 28 post-immunisation, and anti-SARS-CoV-2-S IgG antibody titers were quantified by ELISA. The LOD is indicated by the dotted line.

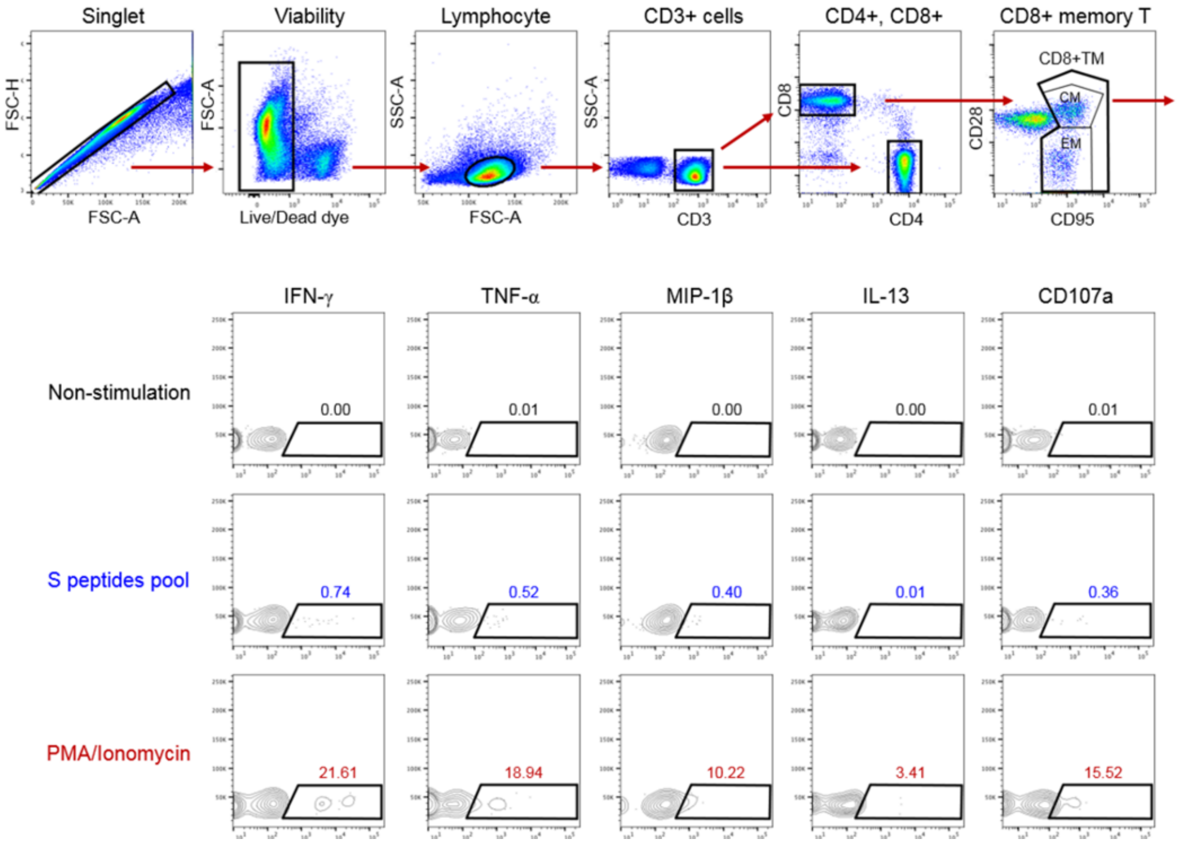

Supplemental figure 2. Intracellular cytokine staining (ICS) gating strategy and representative plots. Flow cytometry gating strategy used to identify antigen-specific total memory CD4$^+$ and CD8$^+$ T cell responses by ICS assay upon stimulation with S peptides pool. A representative gating of CD8+ memory T cells responses is shown. Flow dot plots illustrate individual cytokine with no stimulation, S peptides pool and a positive control PMA/Ionomycin stimulation in each assay.
